# Data mining for the identification of metabolic syndrome status

**DOI:** 10.17179/excli2017-911

**Published:** 2018-01-10

**Authors:** Apilak Worachartcheewan, Nalini Schaduangrat, Virapong Prachayasittikul, Chanin Nantasenamat

**Affiliations:** 1 Department of Community Medical Technology, Faculty of Medical Technology, Mahidol University, Bangkok 10700, Thailand; 2 Department of Clinical Chemistry, Faculty of Medical Technology, Mahidol University, Bangkok 10700, Thailand; 3 Center of Data Mining and Biomedical Informatics, Faculty of Medical Technology, Mahidol University, Bangkok 10700, Thailand; 4 Department of Clinical Microbiology and Applied Technology, Faculty of Medical Technology, Mahidol University, Bangkok 10700, Thailand

**Keywords:** metabolic syndrome, health parameters, diabetes mellitus, cardiovascular diseases, data mining, QPHR

## Abstract

Metabolic syndrome (MS) is a condition associated with metabolic abnormalities that are characterized by central obesity (e.g. waist circumference or body mass index), hypertension (e.g. systolic or diastolic blood pressure), hyperglycemia (e.g. fasting plasma glucose) and dyslipidemia (e.g. triglyceride and high-density lipoprotein cholesterol). It is also associated with the development of diabetes mellitus (DM) type 2 and cardiovascular disease (CVD). Therefore, the rapid identification of MS is required to prevent the occurrence of such diseases. Herein, we review the utilization of data mining approaches for MS identification. Furthermore, the concept of quantitative population-health relationship (QPHR) is also presented, which can be defined as the elucidation/understanding of the relationship that exists between health parameters and health status. The QPHR modeling uses data mining techniques such as artificial neural network (ANN), support vector machine (SVM), principal component analysis (PCA), decision tree (DT), random forest (RF) and association analysis (AA) for modeling and construction of predictive models for MS characterization. The DT method has been found to outperform other data mining techniques in the identification of MS status. Moreover, the AA technique has proved useful in the discovery of in-depth as well as frequently occurring health parameters that can be used for revealing the rules of MS development. This review presents the potential benefits on the applications of data mining as a rapid identification tool for classifying MS.

## Introduction

**Figure 1:**
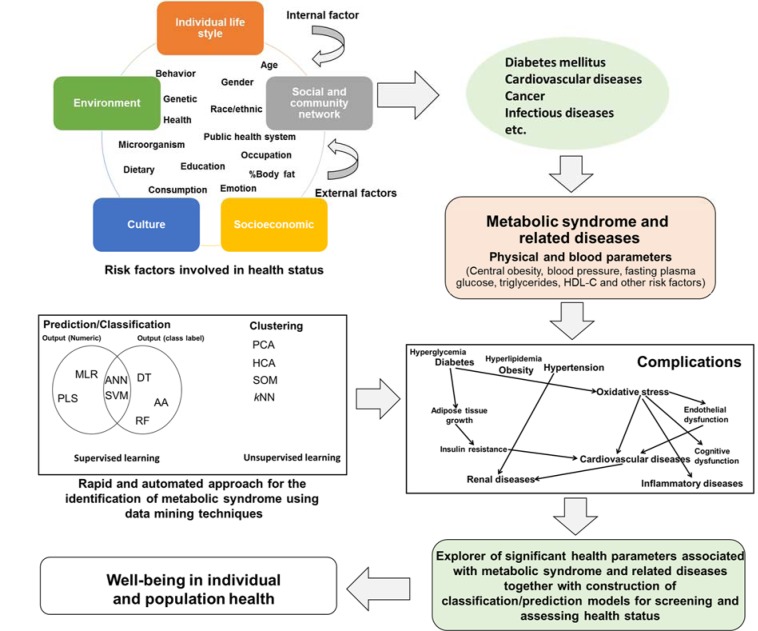
Risk factors of developing diseases and applications of data mining techniques for assessing health status. AA: association rule analysis, ANN: artificial neural network, DT: decision tree analysis, HCA: Hierarchical component analysis, *k*NN: *k*-nearest neighbor, MLR: multiple linear regression, PCA: principal component analysis, PLS: partial least square, RF: random forest, SOM: self-organizing map and SVM: support vector machine

Over the past century, the advents in science and technology have led to significant and enormous changes in the development of countries, economies, societies and environment as well as improving quality of life. However, the effects of these advancements have led to changes and perturbation of individual/population life style, environment, culture, socioeconomic and community network. As a result, this predisposes the population with several internal and external risk factors that possibly cause pathological conditions leading up to diseases (Figure 1(Fig. 1)). These diseases occur via multiple risk factors such as being infected by pathogenic microorganisms (e.g. bacteria, fungi, parasites and viruses), free radicals, carcinogens, toxic compounds, pollutants and genetic abnormalities. Moreover, lifestyle and dietary modifications as well as physical inactivity have led to metabolic abnormalities. The aforementioned risk factors possibly caused diseases such as metabolic syndrome, cardiovascular diseases, diabetes mellitus, cerebrovascular diseases, foodborne diseases, infectious diseases and cancer. Therefore, focusing on health parameters provides an interesting opportunity to explore the health status in individual and population subjects correlating with biochemical changes in the body.

Interestingly, metabolic syndrome (MS) has been implicated in the development of diabetes mellitus (DM) type 2 (WHO, 2008[43]) and cardiovascular disease (CVD) (WHO, 2007[42]). A MS is defined as a clustering of metabolic abnormalities, especially including central obesity (e.g. waist circumference (WC) or body mass index (BMI)), dyslipidemia (e.g. triglyceride (TG) and high-density lipoprotein-cholesterol (HDL-C)), hyperglycemia (e.g. fasting plasma glucose (FPG)), and hypertension (e.g. systolic or diastolic blood pressure (SBP or DBP)) (Alberti et al., 2009[1]). 

The prevalence of DM has been reported in global incidences from 150 million in the year 2000 with a rapid increase to 220 million by 2010 and is estimated to reach 360 million by 2030 (Amos et al., 1997[2]; WHO, 2008[43]). Furthermore, the prevalence of CVD has been predicted to increase from 17.5 million in 2005 to 20 million in 2015 (WHO, 2007[42]). Therefore, the classification of MS for rapid diagnosis to prevent the development of type 2 DM and CVD is urgently required.

The criteria for identifying MS has been developed by many organizations, for example, the first criteria was reported by the World Health Organization (WHO) in 1999 (WHO, 1999[45]). Other criteria for defining MS have been organized by the European Group for the Study of Insulin Resistance (EGIR) (Balkau and Charles, 1999[3]), the National Cholesterol Education Program Adult Treatment Panel III (NCEP ATPIII) (NCEP ATPIII, 2001[28]) and the International Diabetes Federation (IDF) (Alberti et al., 2009[1]). The criteria for identification of MS obtained from different organizations are presented in Table 1(Tab. 1). 

In fact, the geographical location, ethnicity, race as well as various social and dietary behaviors may lead to obesity, hypertension and diabetes. Moreover, according to the IDF criteria, central obesity (i.e. WC or BMI) is usually indicated as the first criteria followed by a set of two or more metabolic abnormalities. The IDF criteria uses BMI in place of waist circumference as it is significantly correlated (Ryan et al., 2008[35]). The cut-off for obesity as outlined by the WHO is BMI ≥ 30 kg/m^2^. However, this value was not appropriate for identifying the BMI status of Asian populations. This may be due to the differences in anthropometry, race/ethnic, percentage of body fat, society and dietary behaviors. Therefore, the cut-off criteria was redefined and constructed by the Steering Committee of the Regional office for the Western Pacific Region of WHO, the International Association for the Study of Obesity and the International Obesity Taskforce (WPRO) to be assigned as the new standard, whereby overweight individuals have a BMI ≥ 23 kg/m^2^ and obese individuals have a BMI ≥ 25 kg/m^2^ (WHO, 2000[44]). Furthermore, Asian populations have a high record of morbidity and mortality rate arising from diabetes mellitus and cardiovascular disease even with a low threshold of central obesity with correspondingly lower waist circumference and lower BMI. Hence, the BMI cut-off for defining obesity in Asian populations was changed to 25 kg/m^2^ (WHO, 2000[44]). This new BMI cut-off has demonstrated successful identification of obesity in the Chinese (Ko et al., 2001[15]), Japanese (Morimoto et al., 2008[23]), Korean (Oh et al., 2004[31]), Taiwanese (Pan et al., 2004[32]) and Thai (Worachartcheewan et al., 2010[47]) populations as well as being used as the first criteria for MS identification. Furthermore, individuals with an abnormal glucose level or a corresponding insulin level as the first component of the WHO and EGIR criteria (Table 1(Tab. 1)), respectively, followed by 2 or more metabolic abnormalities were identified as having MS. In the NCEP ATPIII criteria, individuals having 3 or more of the MS components were defined as having MS (Table 1(Tab. 1)) while the IDF criteria considered the use of the central obesity as the first component followed by 2 or more abnormalities as identification for MS.

## Overview of Data Mining for Assessment of Health Status

### Concepts of data mining

Data mining is the process of analyzing and managing data from a large pool of information which leads to the summarization of the data for obtaining knowledge and insight into large databases which seek unknown patterns, classifications, clustering and relationships in the data set (Han and Kamber, 2001[9]). Data mining is composed of six steps according to the Cross-Industry Standard Process for Data Mining (CRISP-DM) established in 1996. The CRISP-DM aimed to produce a protocol on the performance of data mining that was applicable to everyone (from the novice up to an expert in the field) for a comprehensive data mining methodology and process model (Shearer, 2000[36]). The Knowledge Discovery in Database (KDD) is also used together with data mining. The process of KDD and data mining are similar, however, data mining is one of the steps of the KDD process which includes data selection, data preprocessing, data transformation, data mining, interpretation/evaluation of the model and use of the discovered knowledge (Fayyad et al., 1996[7]).

A typical data set as formatted in a spreadsheet or CSV text file is comprised of patients/individuals (rows) as well as health parameters and class labels (columns). Health parameters are essentially independent variables X_1_*_i_*, X_2_*_i_*,…, X_n_*_i_* defining the unique characteristics of patients/individuals while the class label is a dependent variable Y*_i_*, Y*_ii_*,…, Y_n_*_i_*) of each sample (Nisbet et al., 2009[29]) as shown in Table 2(Tab. 2). 

Prior to model construction, independent variables (quantitative data) are scaled so as to afford comparison of variables by means of normalization or standardization (Nantasenamat et al., 2009[25], 2010[26]).

Normalization for independent variables is adjusted in the range of 0 and 1 according to the following equation:


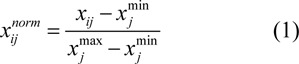


where


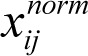


is the normalized value, *x_ij_* is the value of interest,


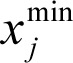


is the minimum value and


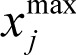


is the maximum value.

Standardization for independent variables is performed in the mean and unit variance by using the following equation:


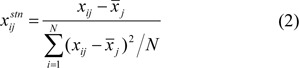


where


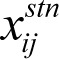


represents the standardized value, *x_ij _*represents the value of each sample, *x̅_j_* represents the mean of each descriptor, and *N* represents the sample size of the data set.

In addition, the original quantitative data (without normalization or standardization) and qualitative data can also be used to directly build predictive models.

In the construction of a predictive model, the data set is typically divided into two sets: 1) training set (i.e. for the training of machine learning algorithms to recognize patterns and generate models) 2) testing set (i.e. for the evaluation of the model). The types of generated testing set can be obtained from internal and external testing sets. Cross-validation is an internal testing set which divides the data set into *n* equal parts whereby one part is used as a testing set and the remaining parts are used as training sets until all parts are used as the testing set. A variety of *n*-fold cross validation selection methods have been used to evaluate the predictive models such as 10-fold cross-validation used for a large number of data set which are generated into 10 parts, for example, 500 subjects were separated into 10 equal parts, where 50 samples were used as the testing set and 450 used as the training set. In contrast, leave-one-out is employed for data sets containing a small number of objects where the numbers of folds are equal to the number of data sets. Furthermore, model validation was also performed using an external set that consists of data not used in the model construction (Nantasenamat et al., 2009[25], 2010[26]).

The types of machine learning are categorized into 2 groups: supervised and unsupervised learning. Supervised learning consists of dependent variables assigned as numerical or class labels that make use of machine learning algorithms for the classification or prediction of the data set whereas unsupervised learning is directly performed on the data set for clustering within which dependent variables are not used (Nantasenamat et al., 2009[25], 2010[26]; Nantasenamat and Prachayasittikul, 2015[27]: Prachayasittikul et al., 2015[33]). Examples of data mining techniques used for supervised and unsupervised learning are displayed in Figure 1(Fig. 1). In supervised learning, the data mining techniques such as MLR, PLS, ANN and SVM are used to construct predictive models in outputs of numeric data as classification and regression models, and DT, AA, RF, ANN and SVM are used for generating classification model in output of class labels. Considering unsupervised learning, the PCA, HCA, SOM, *k*NN clustering and AA, was applied for the build-up of clustering or classifying data in unassigned output data which is used for understanding the distribution of each cluster and for identifying similar or different groups between the information. (Nantasenamat et al., 2010[26]; Nantasenamat and Prachayasittikul, 2015[27]; Prachayasittikul et al., 2015[33]). Each data mining technique has shown its advantage and disadvantage such as ANN and SVM are non-linear techniques as well as black-box methods whereas MLR is an easy technique that is limited in a huge number of features. Therefore, using data mining should be considered with the type of data that can interpret significant parameters related in the output data. Furthermore, data mining could be applied in sciences and health from small molecules, chemical polymer as well as biological macromolecules up to the population level (Isarankura-Na-Ayudhya, 2009[12]). 

### Data mining for medical/clinical applications

Medical/clinical databases are considered as large collections of data composed of patient/individual information such as patient history, physiological and biochemical parameters and diseases which have been collected in the hospital or laboratory systems. Therefore, understanding and revealing relationships using medical/clinical data are needed to obtain new knowledge in medical/clinical fields. Advances in the realm of computational information have allowed the development of new methods and tools for analyzing large quantities of data. Data mining has made use of medical/clinical data for discovering patterns and building predictive models (Iavindrasana et al., 2009[11]; Koh and Tan, 2005[16]; Lee et al., 2000[19]; Obenshain, 2004[30]; Ting et al., 2009[40]; Yoo et al., 2012[51]) to help physicians in the decision-making for diagnosis, prognosis and treatment of patients. In addition, data mining has been successfully applied for identifying and building relationship models to display the relationship between health parameters and diseases such as cancer, cerebrovascular disease, diabetes mellitus, food-borne diseases, heart diseases, hypertension, hyperlipidemia, ischemic heart disease, inflammatory bowel disease and metabolic syndrome as shown in Table 3(Tab. 3) (References in Table 3: Nahar et al., 2011[24]; Yeh et al., 2011[50]; Quentin-Trautvetter et al., 2002[34]; Su et al., 2006[37]; Thakur et al., 2010[39]; Lee et al., 2000[19]; Chang et al., 2011[5]; Wei et al., 2012[41]; Tantimongcolwat et al., 2008[38]; Firouzi et al., 2007[8]; Karimi-Alavijeh et al., 2016[13]; Worachartcheewan et al., 2010[46], 2013[48], 2015[49]).

### Health parameters

Health parameters are important variables for assessing health status and for the proper diagnosis of diseases. These parameters are collected in medical databases and are obtained when individuals receive their health check-up and/or health assessment with disease conditions. Generally, a physician uses blood chemistry and physical examination together with health history and interview in order to evaluate the health status of a patient. However, delaying diagnosis of diseases may lead to morbidity and mortality for the patient. Therefore, the progression of informative computational technology can help physicians rapidly diagnose and find patterns that recognize risk factors related to developing diseases. As mention above, medical databases collecting a large amount of data are interesting and can be used as a health status evaluation of diseases for individuals. Therefore, to manage this data, powerful computational tools are necessary. Particularly, machine learning approaches namely, data mining is applied on health parameters as to discover patterns and construct predictive models of diseases. The benefit of data mining, using biomedical databases, is for the rapid and automatic diagnosis of MS in order to help with therapeutic or health prevention for individuals having risk factors for disease development.

### Statistical analysis

To evaluate predictive models, the statistical parameters were performed which comprised of accuracy, sensitivity, specificity, positive predictive value (PPV), negative predictive value (NPV) (Kuo et al., 2001[17]) and Matthews correlation coefficient (MCC) (Matthews, 1975[21]). These statistical parameters are calculated using the following equations:


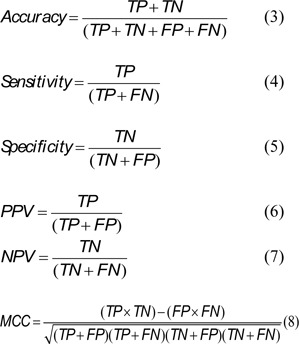


where TP is the number of true positives, TN is the number of true negatives, FP is the number of false positives or over-predictions and FN is the number of false negatives or missed predictions. The value of MCC is 0 for a random assignment and 1.0 for a perfect prediction (Matthews, 1975[21]).

## Quantitative Population-Health Relationship (QPHR)

The utilization of data mining techniques for assessing the health status in a population via their health parameters had previously been termed by us as quantitative population-health relationship (QPHR) (Worachartcheewan et al. 2013[48]). QPHR makes use of data mining to elucidate the relationship between physical and biochemical parameters from populations/patients with diseases using data mining technique.

Data mining has been used to extract and explore knowledge from a large amount of data in clinical/medicinal settings. A variety of data mining techniques including SVM, ANN, MLR, PCA, SOM, DT and AA have been demonstrated for constructing predictive models of diseases (Chang et al., 2011[5]; Firouzi et al., 2007[8]; Kim et al., 2012[14]; Lee et al., 2000[19]; Nahar et al., 2011[24]; Worachartcheewan et al., 2010[47][46], 2013[48], 2015[49]; Yeh et al., 2011[50]). In addition, data mining has previously been employed to generate QSAR/QSPR models for insight into correlations between physicochemical descriptors and their biological/chemical properties (Nantasenamat et al., 2009[25], 2010[26]; Nantasenamat and Prachayasittikul, 2015[27]; Prachayasittikul et al., 2015[33]).

**Figure 2:**
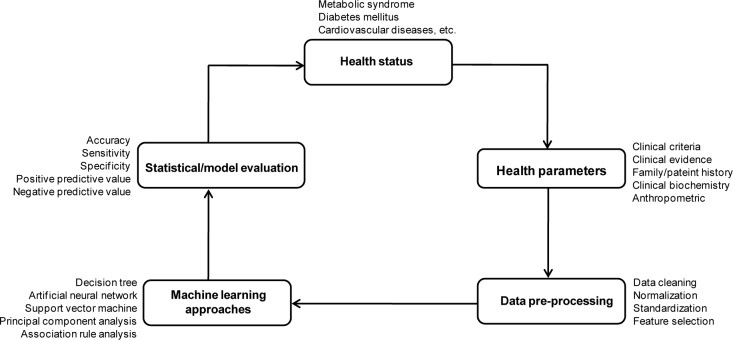
Schematic representation of the QPHR models

QPHR models were used to discover unknown or hidden parameters associated with the progression of diseases. The QPHR models are performed with a clinical aim in diagnosis, prevention and health promotion of populations/patients. Furthermore, the QPHR models could be useful in medical/clinical data for identifying important risk factors of diseases and classifying individuals who have risk factors in development of said diseases. The procedure of QPHR is illustrated in Figure 2(Fig. 2).

The concept of QSAR/QSPR and QPHR is similar as they are both used in the construction of predictive models for biological/chemical properties (Nantasenamat et al., 2009[25], 2010[26]; Nantasenamat and Prachayasittikul, 2015[27]) and diseases (Worachartcheewan et al., 2013[48]), respectively. In QSAR/QSPR models, quantum chemical and molecular descriptors with their bioactivities are used to find relationships between physicochemical properties and their activities while in QPHR models, health parameters (physiological and blood chemical testing) with diseases are employed to discover patterns or elucidate the relationships between them (Table 4(Tab. 4)).

The QPHR models could easily be adapted for identifying the development of other diseases. Therefore, QPHR can be used to discover unknown or hidden parameters associated with the progression of diseases for the diagnosis, prevention and health promotion in populations/patients.

In this review, examples of QPHR investigations on MS identification were described and demonstrated. In addition, Figure 1(Fig. 1) displayed the application of data mining techniques which discovered important health parameters as well as risk factors associated with MS and related diseases together with the construction of classification/prediction models for screening and assessing health status leading to increased the well-being in individuals and population health. 

MS has been focused as a risk factor associated with DM and CVD. The main cause of MS includes metabolic abnormalities in protein, carbohydrate and lipid metabolisms. Considering the MS criteria, central obesity (BMI/WC), hypertension (SBP or DBP), dyslipidemia (TG and HDL-C) and hyperglycemia (FPG) are integral component that define MS (Table 1(Tab. 1)). Furthermore, unknown components correlating with MS have been discovered whereby other factors involved in MS such as genes, socioeconomic status, behavior and dietary intake were demonstrated. In addition, the in-depth components of health parameters that occur frequently together were also illustrated using data mining. Applications of medical data mining for the classification of MS is essential for the early detection before individuals with high risk factors develop DM and CVD.

### MS classification using various machine learning approaches

Data mining has been employed to identify MS using various approaches such as ANN, SVM, RT, DT and PCA (Worachartcheewan et al., 2013[48]; de Edelenyi et al., 2008[6]). In addition, AA technique is also used for discovering combinations of metabolic abnormalities of MS that occur frequently together. The AA rule is correlated with the previous studies that involved metabolic abnormalities based on high levels of TG, FPG and BP and low level of HDL-C (Worachartcheewan et al., 2010[47][46]; Lee et al., 2008[18]). Moreover, the term of applying data mining for assessing health status via health parameters has been organized and called QPHR by Worachartcheewan et al. (2013[48]). QPHR is defined by using health parameters for the identification associated with health status or diseases that can provide insight into the relationship between an individual's health parameters and the development of diseases. In correlating health parameters with MS status, several machine learning techniques have previously been employed, which comprises of ANN, SVM, DT and PCA (Worachartcheewan et al., 2013[48]). Classification models for MS using various multivariate analysis have been reported that DT is the best QHPR method outperforming ANN and SVM with correct classification of MS and non-MS in greater than 99 % of cases, followed by ANN and SVM displaying an accuracy of more than 98 % and 91 % (Worachartcheewan et al., 2013[48]), respectively. PCA is used for clustering analysis that displays distinctive MS and non-MS groups. The AA gave the rules that provide health parameters with abnormalities of MS component occurring frequently together (Worachartcheewan et al., 2013[48]). In addition, an in-depth analysis for the identification of MS component combinations were explored using AA in order to discover metabolic abnormalities of MS components occurring frequently together. The AA was performed by stratified data from quantitative data to qualitative data using WHO and IDF criteria of metabolic abnormalities. This finding showed the combinations of MS components corresponding to previous studies and obtained association rules for the definition of MS (Worachartcheewan et al., 2010[47][46]; Lee et al., 2008[18]). This work was studied in the Thai population.

Interestingly, DT has been applied to find MS components in the urban and rural Korean population (Kim et al., 2012[14]). The MS was identified using Modified National Cholesterol Education Program Adult Treatment Panel III criteria. DT displayed the combinations of high TG + high SBP, high TG + low HDL-C and high WC + high SBP + high FPG for MS in the urban population while TG + SBP + WC and SBP + WC + FPG for MS in the rural population. From this result, similar patterns for combinations of MS components were observed in the previous study and were highlighted by our results (Worachartcheewan et al., 2010[47][46]).

In addition, DT analysis is considered to be a robust data mining technique for constructing predictive model of metabolic syndrome status with accuracy of 73.90 % (Kim et al., 2012[14]) and 99.86 % (Worachartcheewan et al., 2010[46], 2013[48]). Furthermore, the SVM method has been shown to yield accuracy of 75.70 % (Karimi-Alavijeh et al., 2016[13]) and 91.98% (Worachartcheewan et al., 2013[48]). Moreover, the CHAID decision tree has been shown to display an accuracy of 71.80 % for identifying MS. It was found that WC, TG, HDL-C, and FPG were significant health parameters for the prediction of MS (Miller et al., 2014[22]).

The AA has been used to find patterns of MS related diseases. The study conducted on Taiwanese population by Chan et al. (2008[4]) in MS and DM patients using AA, discovered the relationship between the diseases. It was observed that individuals having high MS were correlated with liver disease and DM individuals were associated with oral diseases such as dental carries, pulpitis, acute gingivitis and periodontosis. Thus, the AA technique exhibited the rules of relations between diseases that can be used to help diagnosis in order to prevent illnesses in patients.

Furthermore, this technique was used to explore association rules between MS and lifestyle (Huang, 2013[10]). It was found that individuals having a BMI >27 kg/m^2^ and/or participating in vigorous physical exercise less than once a week were predisposed to having MS.

In addition, ANN and multiple logistic regression have been employed for identifying MS in patients treated with second-generation antipsychotics (SGAs) (Lin et al., 2010[20]). The results indicated that ANN and logistic regression models gave high accuracy of 88.3 and 83.6%, respectively, while WC, BMI, DBP and gender were important variables for identifying MS in patients undergoing SGA treatment.

A study conducted on the French population by de Edelenyi et al. (2008[6]) showed factors or combinations of factors associated with MS. Particularly, RF was applied for predicting the MS status. Dietary and genetic parameters were used as independent variables while MS or non-MS classes were used as the dependent variables. Important variables were deduced from RF including plasma concentrations of palmitoleic acid, gamma-linolenic acid (GLA) and linoleic acid. Furthermore, 3 essential single-nucleotide polymorphisms (SNPs) were selected by RF composed of APOB rs512535, LTA rs915654 and ACACB rs4766587. The correct classification is 71.4% to predict the MS status. For interpretation of health parameters, it showed that the palmitoleic acid was significantly higher in MS than non-MS while APOB rs512535 A>G and ACACB rs4766587 A>G correlated with the development of MS. Furthermore, the RF method was used to explore important health parameters and identify MS by Worachartcheewan et al. (2015[49]). It was found that TG is considered as the first significant health parameter associated with MS and gave an accuracy > 98 % for the classification of MS. These results correlated with the previous study (Worachartcheewan et al., 2010[46]).

The examples of data mining application techniques are used for the classification or identification of MS. These examples help to identify patterns of MS component combinations and find the rules of metabolic abnormalities and related diseases associated with MS.

Furthermore, in this review, MS has been focused on risk factors associated with DM and CVD. MS is associated with metabolic abnormalities in protein, carbohydrate and lipid metabolisms. Concerning MS criteria, central obesity (BMI/WC), BP, dyslipidemia (TG and HDL-C) and hyperglycemia (FPG) are components that can be used to define MS. Furthermore, unknown components correlated with MS have been discovered in order to find other factors that are involved such as genes, socioeconomic status, behavior and diet. In addition, an in-depth analysis of components occurring frequently together was demonstrated via data mining. The application of medical data mining to classify MS is an essential performance for early detection of DM and CVD. Therefore, the data mining could be recommended for identification of MS during an individual's health assessment.

A summary of examples employing data mining for the classification of MS is presented in Table 5(Tab. 5) (References in Table 5: de Edelenyi et al., 2008[6]; Karimi-Alavijeh et al., 2016[13]; Kim et al., 2012[14]; Chan et al., 2008[4]; Huang, 2013[10]; Lin et al., 2010[20]; Worachartcheewan et al., 2010[46], 2013[48], 2015[49]; Miller et al., 2014[22]). It was used to identify patterns or combinations of MS components as well as to deduce rules for metabolic abnormalities associated with MS.

## Conclusion

This review article represents the first work of its kind whereby a summary of data mining for the assessment of MS status and discovery of in-depth MS components has been portrayed. This article summarizes the utilization of data mining techniques as a rapid identification tool for the classification of MS and non-MS categories. Complementary knowledge gained from association analysis provides pertinent information on frequently occurring parameters for defining MS. Furthermore, decision tree analysis offers insights on rules leading up to MS or non-MS groups. The topics covered in this article represent an exciting and growing area whereby various machine learning techniques offer useful insights in unravelling the mechanistic basis for MS.

The applications of data mining for the identification of MS and non-MS have been demonstrated and could potentially be employed as a rapid identification tool for classifying MS. Furthermore, association rule analysis was able to discover the important rules for defining MS. In addition, DT has been shown to be a robust machine learning approach for classifying MS and therefore holds great potential for assessing an individual's risk of MS.

## Acknowledgements

This research project is supported by the Office of the Higher Education Commission and Mahidol University under the National Research Universities Initiative and the research grant of Mahidol University (B.E. 2556-2558).

## Figures and Tables

**Table 1:**
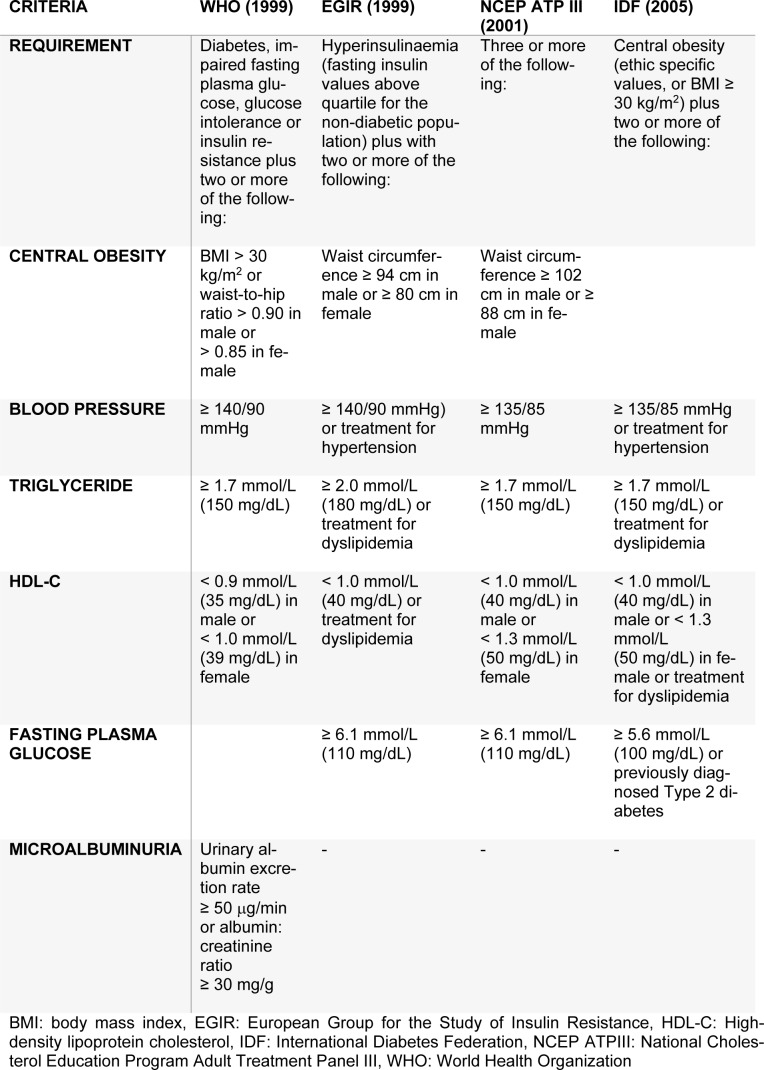
Criteria for defining metabolic syndrome

**Table 2:**
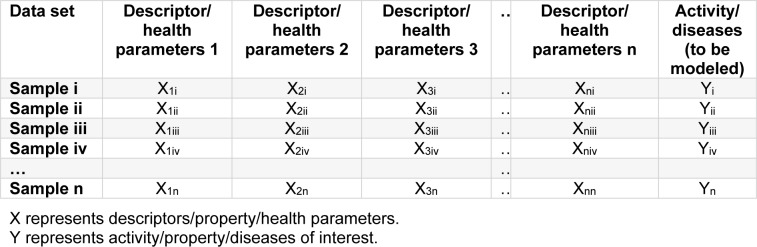
Typical data set format for data mining

**Table 3:**
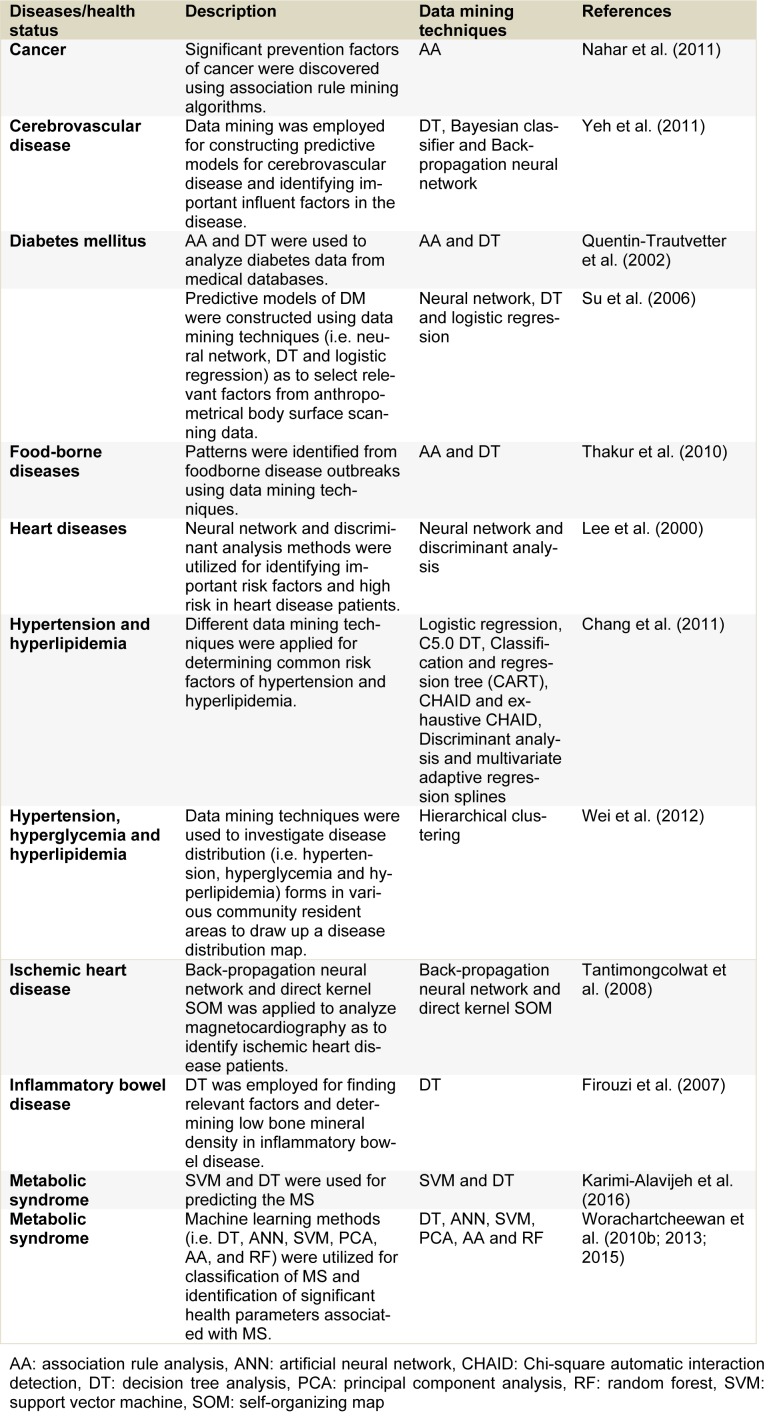
Example of applications of data mining for medical/clinical data

**Table 4:**
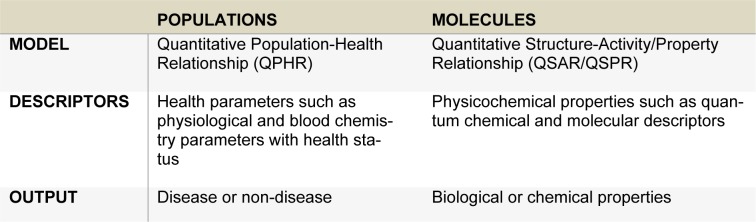
The concept of QPHR and QSAR/QSPR models

**Table 5:**
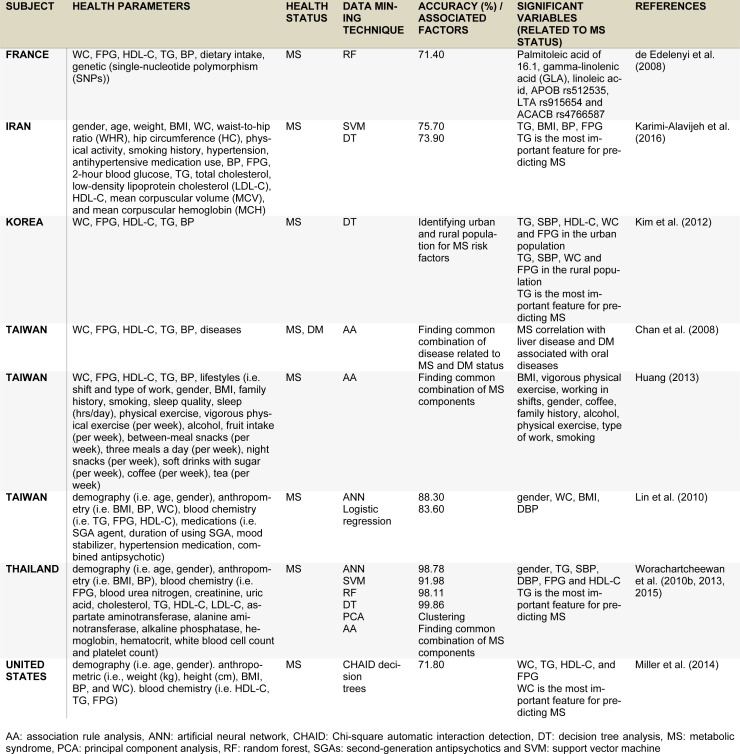
Summary of identifying MS using data mining techniques
